# Use of Nanotrap particles for the capture and enrichment of Zika, chikungunya and dengue viruses in urine

**DOI:** 10.1371/journal.pone.0227058

**Published:** 2020-01-07

**Authors:** Shih-Chao Lin, Brian D. Carey, Victoria Callahan, Ji-Hyun Lee, Nicole Bracci, Anurag Patnaik, Amy K. Smith, Aarthi Narayanan, Benjamin Lepene, Kylene Kehn-Hall

**Affiliations:** 1 National Center for Biodefense and Infectious Diseases, School of Systems Biology, George Mason University, Manassas, VA, United States of America; 2 Ceres Nanosciences Inc., Manassas, VA, United States of America; 3 School of Systems Biology and Computational Materials Science Center, George Mason University, Manassas, VA, United States of America; CEA, FRANCE

## Abstract

Nanotrap^®^ (NT) particles are hydrogel microspheres developed for target analyte separation and discovery applications. NT particles consist of cross-linked N-isopropylacrylamide (NIPAm) copolymers that are functionalized with a variety of chemical affinity baits to enable broad-spectrum collection and retention of target proteins, nucleic acids, and pathogens. NT particles have been previously shown to capture and enrich arboviruses including Rift Valley fever and Venezuelan equine encephalitis viruses. Yet, there is still a need to enhance the detection ability for other re-emerging viruses such as Zika (ZIKV), chikungunya (CHIKV), and dengue (DENV) viruses. In this study, we exploited NT particles with different affinity baits, including cibacron blue, acrylic acid, and reactive red 120, to evaluate their capturing and enrichment capability for ZIKV, DENV and CHIKV in human fluids. Our results demonstrate that CN1030, a NT particle conjugated with reactive red 120, can recover between 8-16-fold greater genomic copies of ZIKV, CHIKV and DENV in virus spiked urine samples via RT-qPCR, superior to the other chemical baits. Also, we observed that CN1030 simultaneously enriched ZIKV, CHIKV and DENV in co-infection-based settings and could stabilize ZIKV, but not CHIKV infectivity in saliva spiked samples. CN1030 enriched viral detection at various viral concentrations, with significant enhancement observed at viral titers as low as 100 PFU/mL for ZIKV and 10 PFU/mL for CHIKV. The detection of ZIKV was further enhanced with NT particles by processing of larger volume urine samples. Furthermore, we developed a magnetic NT particle, CN3080, based on the same backbone of CN1030, and demonstrated that CN3080 could also capture and enrich ZIKV and CHIKV in a dose-dependent manner. Finally, *in silico* docking predictions support that the affinity between reactive red 120 and ZIKV or CHIKV envelope proteins appeared to be greater than acrylic acid. Overall, our data show that NT particles along with reactive red 120 can be utilized as a pre-processing technology for enhancement of detecting febrile-illness causing viruses.

## Introduction

Nanotrap® (NT) particles are cross-linked N-isopropylacrylamide (NIPAm) copolymers that are functionalized with various chemical affinity baits that aid in the collection and binding of target analytes that may range from peptides, proteins, nucleic acids and whole pathogens. NT particles contain a copolymer structure that is thermostable and capable of aiding in retention of target analytes in varying biological conditions such as temperature or pH variable conditions [1, 2]. Additionally, selectivity of NT particles is maintained through the use of chemically fixed affinity baits that consist of reactive dyes such as reactive red, cibacron blue or acrylic acid affinity bait residues [3]. These residues are covalently coupled to the NT core and provide broad spectrum retention of analytes. In addition to maintaining a copolymer core, some versions of NTs contain hydrogel shells functionalized with sulfonic acid. The microspheres containing sulphonic acid shells aid in decreasing non-specific absorption of albumin, enhancing the affinity of NT particles to their targeted analyte [4].

Previously, we have applied NT particles to efficiently capture various viral pathogens such as human immunodeficiency virus (HIV), Rift Valley fever virus (RVFV) and influenza viruses [5–8] and have shown the detection levels of these viruses could be enhanced in various matrices. Due to the spread of arthropod-borne viruses and re-emerging viruses such as Zika virus (ZIKV), dengue virus (DENV) and chikungunya virus (CHIKV) [9], it is necessary to develop innovative and alternative testing methods with higher sensitivity to detect viral particles in body fluids. The Centers for Disease Control and Prevention (CDC) has set guidelines for laboratory confirmation of infection. The current method is to detect viral nucleic acid and/or ZIKV IgM antibody testing in specimens followed by confirmation via plaque reduction neutralization (PRNT) assay toward equivocal serum. For example, subjects who exhibit positive results of ZIKV nucleic acid testing on both urine and serum are considered as a positive for ZIKV infection. If the nucleic acid testing is negative, but the patient tests positive for ZIKV IgM, then the PRNT assay will need to be applied to confirm infection [10]. Therefore, enhancing viral nucleic acid testing is an important step to improving viral diagnostics.

Here, we extend our previous work on NT particles to determine if they can be utilized for improved detection of Flaviviruses (ZIKV and DENV) and an additional alphavirus (CHIKV). As ZIKV, CHIKV and DENV are detectable in patients’ urine samples by RT-qPCR [11–13], we assessed the ability of NT particles to efficiently capture and enrich viruses in urine. Our previous work with NT particle capture of viruses has been performed in serum, saliva or nasal fluid [3, 6]. The ability of NT particles to function in co-infection models as well as the limitation-of-detection of RT-qPCR methods with NT particles were determined. Finally, we evaluated the utilization of a magnetic version of NT particles that simplifies the sample processing protocol and provides opportunities for improved workflows and integration into collection devices and point-of-care instruments.

## Materials and methods

### Viral strains, NT particles, urine and saliva

ZIKV-MR766 was obtained from BEI Resources (catalog numbers NR-50065). DENV-2 was obtained from American Type Culture Collection (ATCC-VR-1810). The vaccine strain of CHIKV (181/25) was kindly provided by Dr. Naomi Forrester, University of Texas Medical Branch, Galveston [14]. Standard and magnetic versions of NT particles coated with 3 different affinity baits (Table 1) were provided by Ceres Nanosciences. The human urine and saliva were directly purchased from Bioreclamation and stored in -20°C. Prior to experiments, urine was thawed and filtered with a 0.22 μM filter and saliva was diluted by Puritan® UniTranz-RT Sterile Universal Transport Solution in a 1:3 ratio before spiking viruses.

**Table 1:** Nanotrap® particle indicators with shell, core and affinity bait details for standard and magnetic particles.

Particle Name	Type	Shell Detail	Core Detail	Affinity Bait
CN1030	Standard Hydrogel	No shell	N-isopropylacrylamide (NIPAm)	Reactive red 120
CN2030	Standard Hydrogel	Shell	N-isopropylacrylamide (NIPAm)	Acrylic acid
CN2010	Standard Hydrogel	Shell	N-isopropylacrylamide (NIPAm)	Cibacron blue F3GA
CN3070	Magnetic	No shell	N-isopropylacrylamide	Reactive red 120
CN4000	Standard Hydrogel Mixture	Mixture	1:1:1 mixture of CN1030, CN2030, CN2010	Contains all three affinity bait-types

### Virus processing method by NT particles

Viruses were spiked directly into urine at a final titer of 1 × 10^6^ PFU/mL unless otherwise noted. One hundred microliters of NT particles were added to 1 mL of sample unless otherwise noted. Samples were incubated at room temperature on a tube rotator for 30 min followed by either centrifugation at 13,200 rpm at 4°C (standard particle) for 30 min or separated by a spin magnet base (magnetic particle) for 5 min at room temperature. The supernatant was removed and the pellet was resuspended in ddH_2_O before further experiments.

For co-infection experiments, the two selected viruses were spiked together into urine at the same viral titers (1 × 10^6^ PFU/mL) followed by the same NT processing procedures described above.

### RNA extraction and RT-qPCR

Total viral RNA was isolated by using RNeasy Mini kit (Cat#74104, Qiagen, German) as instructed by manufacturer’s manual. RT-qPCR was performed by RNA UltraSense One-Step Quantitative RT-PCR reagents (Cat# 1173927, ThermoFisher, USA) and StepOne^™^ Real-Time PCR System (Cat# LS4376357, Applied Biosystems, USA) along with virus-specific primers and probes as below:

ZIKV [15]:

Forward: CCG CTG CCC AAC ACA AG;

reverse: CCA CTA ACG TTC TTT TGC AGA CAT;

Probe: FAM- AGC CTA CCT TGA CAA GCA GTC AGA CAC TC

CHIKV [16]:

Forward: CAT CTG CAC YCA AGT GTA CCA;

Reverse: GCG CAT TTT GCC TTC GTA ATG;

Probe: FAM- GCG GTG TAC ACT GCC TGT GAC YGC

DENV-2:

Forward: CTG CAR GGA CGA GGA CCA TTA;

Reverse: GGG ATT GTT AGG AAA CGA AGG A;

Probe: JOE- TTG AAA CTG TTC ATG GCC CTG GTG

Published protocols for RT-qPCR were utilized [15–17]. Viral genomic copies were determined by comparing the unknowns to a standard curve containing known RNA quantities and extrapolating the value. RNA standards were made by extracting viral RNA from ZIKV, CHIKV, and DENV viral supernatants using TRIzol LS Reagent and quantifying the viral RNA with Quant-iT Ribogreen RNA assay kit (Thermo-Fisher Scientific).

### Prediction of protein-ligand docking

CHIKV envelope glycoprotein heterodimer (PDB ID: 3N43) and ZIKV E homodimer protein (PDB ID: 5JHM) coordinate files were obtained from PDB website and the chemical structures of affinity baits reactive red 120, cibacron blue, and acrylic acid were downloaded from PubChem. The docking predictions were performed with AutoDock v4.2.6 [18] and with the SwissDock website (http://www.swissdock.ch) [19] with native binding modes (NBM), and predicted docking results were visualized and analyzed with UCSF Chimera (v1.13.) [20]. For ZIKV docking model, only one chain of E protein homodimer was used.

### Statistical analysis

Non-parametric statistics, Mann-Whitney U test and Kruskal-Wallis test, were performed throughout this study to calculate the statistical significance among groups unless indicated elsewhere.

## Results

### Screening of standard NT particles for capture and enrichment of arboviruses in urine

**Fig 1:**
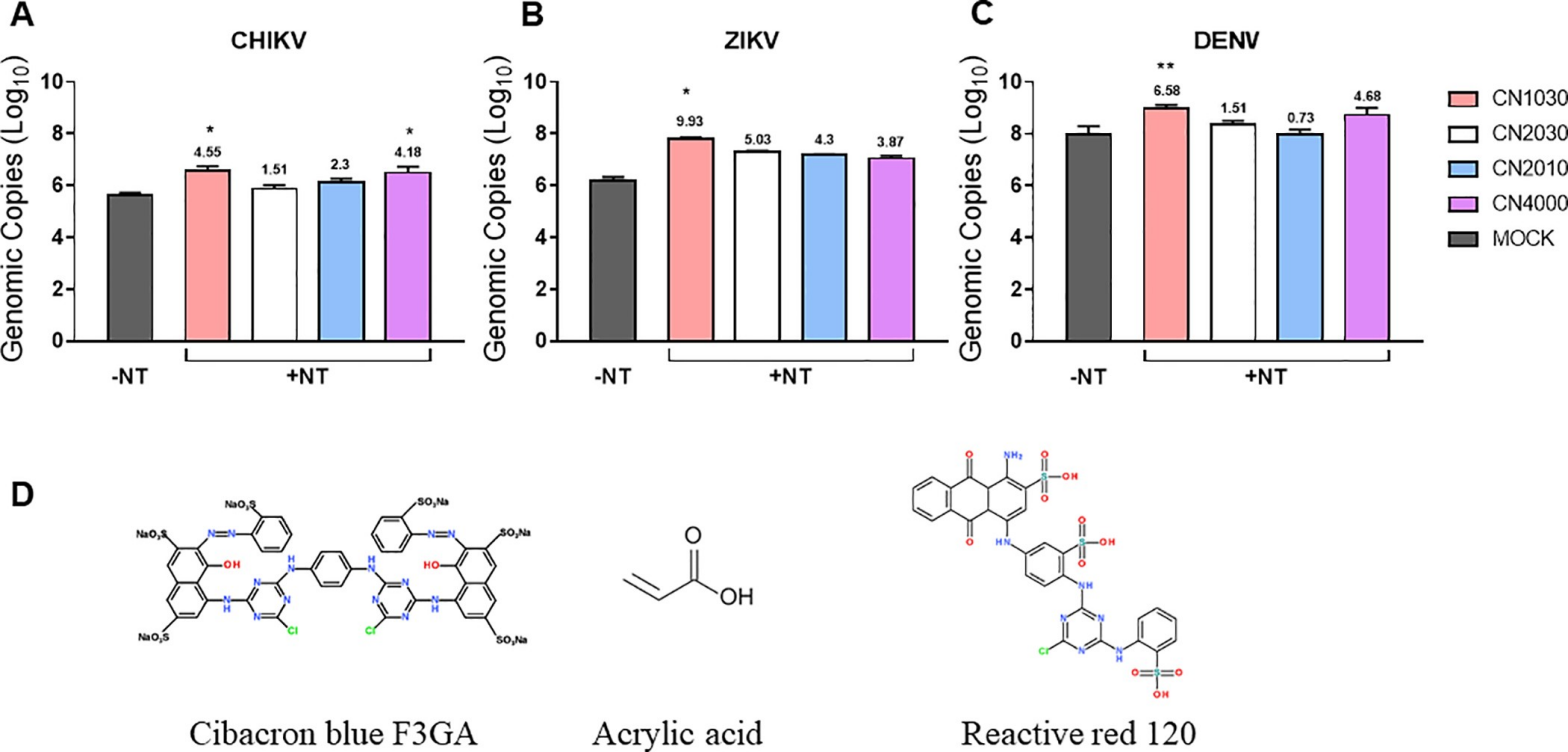
Screening of NT particles for febrile illness pathogen enrichment. (A) CHIKV, (B) ZIKV and (C) DENV were spiked into urine and incubated with CN1030, CN2030, CN2010 or CN4000. Viral RNA was extracted and quantitate by RT-qPCR. Samples without NT particles (-NT) are included as controls. Numbers above the +NT bars indicate the fold-change enrichment based on the following calculating formula: genomic copies of NT positive were divided by those in Nanotrap negative (no-NT control) groups. (D) The diagrams of chemical structures of three affinity baits. Data was presented as mean ± SEM from three or four biological replicates. *P* value is ≤ 0.05; ** *P* ≤ 0.01.

Previous work with NT particles has shown that particles containing cibacron blue, reactive red, or acrylic acid affinity baits are the most effective at enhancing detection of enveloped viruses [6–8]. Therefore, we tested NT particles containing these three baits as well as a NT particle containing a combination of all 3 baits (Table 1) for their ability to capture and enrich CHIKV, DENV and ZIKV from human urine. The viruses were spiked in human urine at a viral titer of 1 × 10^6^ PFU/mL and the enrichment efficiency of NTs determined based on the viral genomic copies obtained from RT-qPCR in the presence or absence of NT. CN1030 with reactive red 120 exhibited the best capture efficiency, enriching all viruses tested most efficiently (Fig 1). CHIKV was enriched by 4.6-fold, ZIKV by 9.9-fold and DENV by 6.6-fold. CN2030 with acrylic acid showed limited capturing capability of CHIKV and DENV (1.5-fold enrichment) but not ZIKV (5-fold enrichment observed) Notably, CN4000 only contains a one-third proportion of CN1030, but it still exhibited effective, albeit reduced, capturing ability for all three viruses (~4-fold enrichment for each virus).

### CN1030 captures and enriches DENV, CHIKV, and ZIKV in co-infection model systems

**Fig 2:**
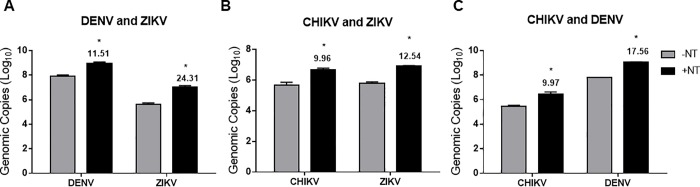
CN1030 captures and enriches DENV, CHIKV, and ZIKV in co-infection model systems. The viral preference for enrichment of CN1030 (+NT) was investigated in co-infection models. 10^6^ PFU/mL of (A) DENV and ZIKV, (B) CHIKV and ZIKV, and (C) CHIKV and DENV were spiked in urine and incubated with (+NT) or without CN1030 (-NT) followed by quantification of viral RNA by RT-qPCR. Numbers above the +NT bars indicate the fold-change enrichment generated by dividing with genomic copies in no-NT controls. Data was shown as mean ± SEM from four biological replicates where significance was indicated as *, *P* <0.05.

Given that cases of simultaneous infection of multiple arboviruses have been reported [21, 22], we next aimed to test if CN1030 is able to enrich viruses when two different viruses were mixed in one sample. We spiked DENV and ZIKV, CHIKV and ZIKV, or CHIKV and DENV into urine samples at the same viral titer followed by incubation with CN1030. We found that CN1030 can overall enrich each virus in all three co-infection models (Fig 2). CN1030 enriched more of ZIKV from the DENV-ZIKV model than that in the CHIKV-ZIKV model (Fig 2A and 2B). A similar pattern was observed with DENV, where more DENV was enriched in the CHIKV-DENV model than in the DENV-ZIKV model (Fig 2A and 2C). The fold of enrichment for CHIKV in both models (CHIKV-ZIKV and CHIKV-DENV) remained approximately the same (Fig 2B and 2C).

### NT particles preserves ZIKV infectivity

**Fig 3:**
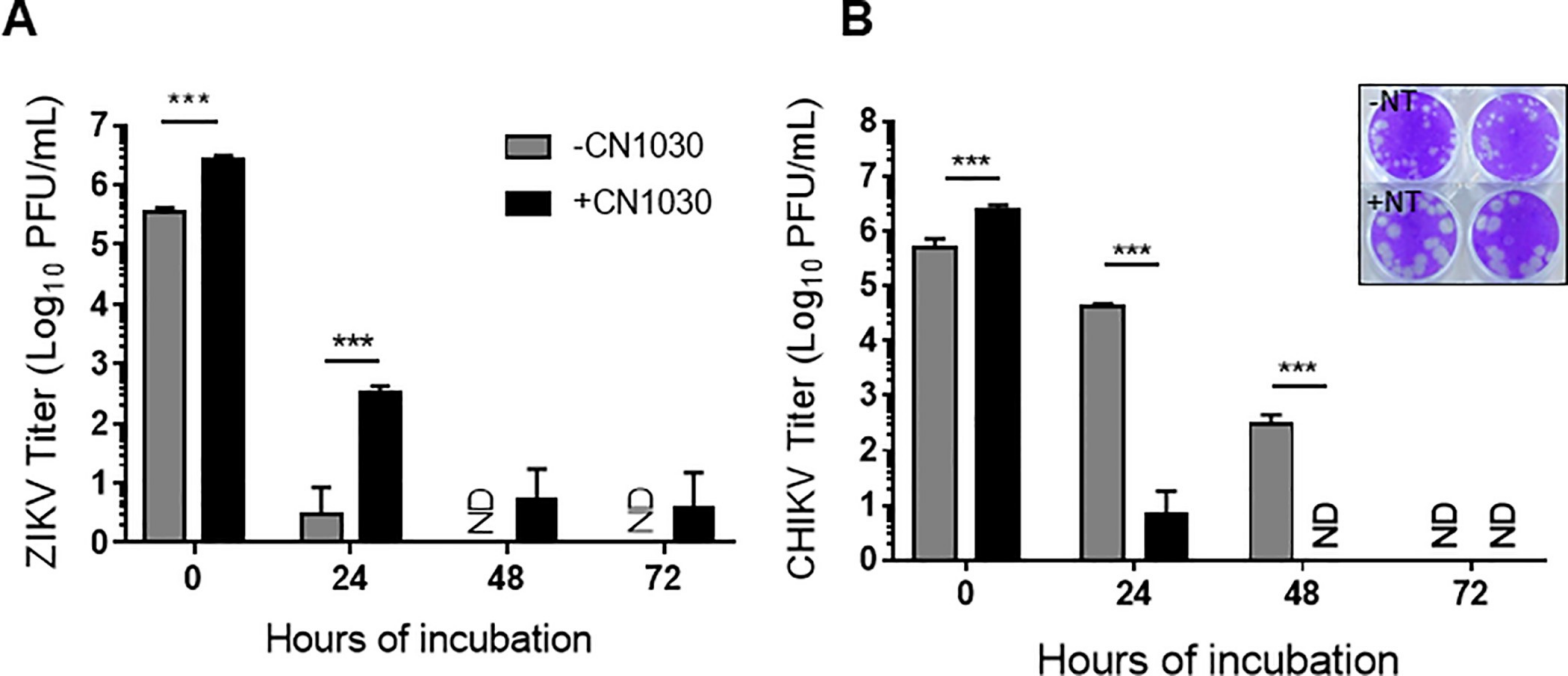
NT particles preserves ZIKV infectivity. 10^6^ PFU/mL of (A) ZIKV and (B) CHIKV were spiked in human saliva with or without CN1030 incubation in 1:10 ratio (CN1030: saliva-spiked virus) for 30 min at ambient temperature. Viral titers were subsequently determined by plaque assays at 0, 24, 48, and 72 hours post-incubation at 37°C. Inserted images in panel B show the plaque morphology and sizes of CHIKV. ND, not detected. Data were presented as mean ± SEM of at least three biological replicates and analyzed with two-way ANOVA (***, *P*<0.001).

Previous work found that NT particles preserved the infectivity of RVFV at elevated temperatures [8]. Since CN1030 demonstrated the best efficiency in capturing the tested viruses, we investigated whether infectious virions can also be preserved by CN1030. Experiments indicated that no infectious virions could be obtained in ZIKV-spiked urine samples. As ZIKV and CHIKV can also be detected in patients’ saliva [12, 23], we used saliva as an alternative sample matrix. Saliva samples spiked with ZIKV or CHIKV were incubated at 37°C with and without CN1030 for 0, 24, 48, and 72 h. At 0h CN1030 was found to enrich infectious ZIKV by ~1 log (Fig 3A). In the absence of CN1030, viral titers dropped sharply at 24 (~10 pfu/ml detected) and no plaques were detected at 48 and 72 h. In contrast, infectious ZIKV was detected at all times in samples containing CN1030, albeit at significantly reduced titers. On the contrary, despite the enrichment of infectious CHIKV at 0 h, we did not observe the preservation of CHIKV (Fig 3B). Rather, we detected significantly less infectious CHIKV at 24 and 48 h post-incubation. Interestingly, we noticed that the sizes of CHIKV plaques were much bigger when incubated with CN1030, so the reduction of viral titers may be due to the technical limitation of plaque assay being unable to distinguish aggregated viral particles (Fig 3B, insert).

### NT particles enhance ZIKV and CHIKV detection at both low and high titers

**Fig 4:**
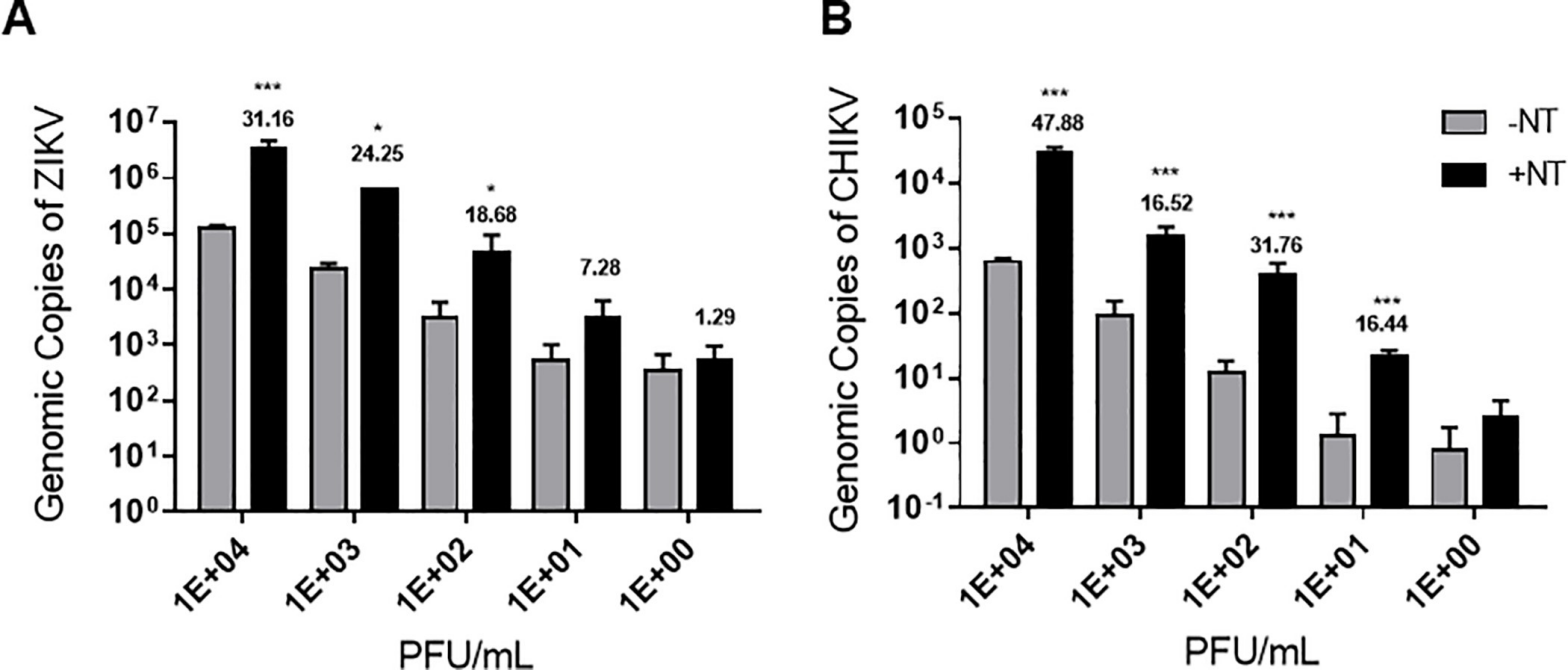
NT particles enhance ZIKV and CHIKV detection at both low and high titers. Detection of ZIKV (panel A) or CHIKV (panel B) by RT-qPCR with or without CN1030 was evaluated through the use of decreasing concentrations of viruses (PFU/mL) spiked in urine. Numbers above the +NT bars indicate the fold-change enrichment. Data are shown as mean ± SD for three biological replicates. The statistical significance was performed with two-way ANOVA and marked with an asterisk (*) symbol where *** indicates *P* value is ≤ 0.001.

We further evaluated the LOD of CHIKV and ZIKV in the presence or absence of CN1030. To this end, we diluted spiked viruses in a 10-fold manner and incubated samples in the presence or absence of CN1030 prior to RT-qPCR. Our results indicate that CN1030 was capable of significantly enhancing the detection levels of ZIKV RNA at 10^4^, 10^3^, 10^2^, but not 10 and 1 PFU/mL of spiked titers compared to samples without NT particles (Fig 4A). Similar results were obtained with CHIKV with enhanced detection observed at 10^4^, 10^3^, 10^2^, 10, but not 1 PFU/mL. Hence, our data demonstrate that CN1030 could efficiently enrich ZIKV down to 100 PFU/mL and CHIKV down to 10 PFU/mL.

**Fig 5:**
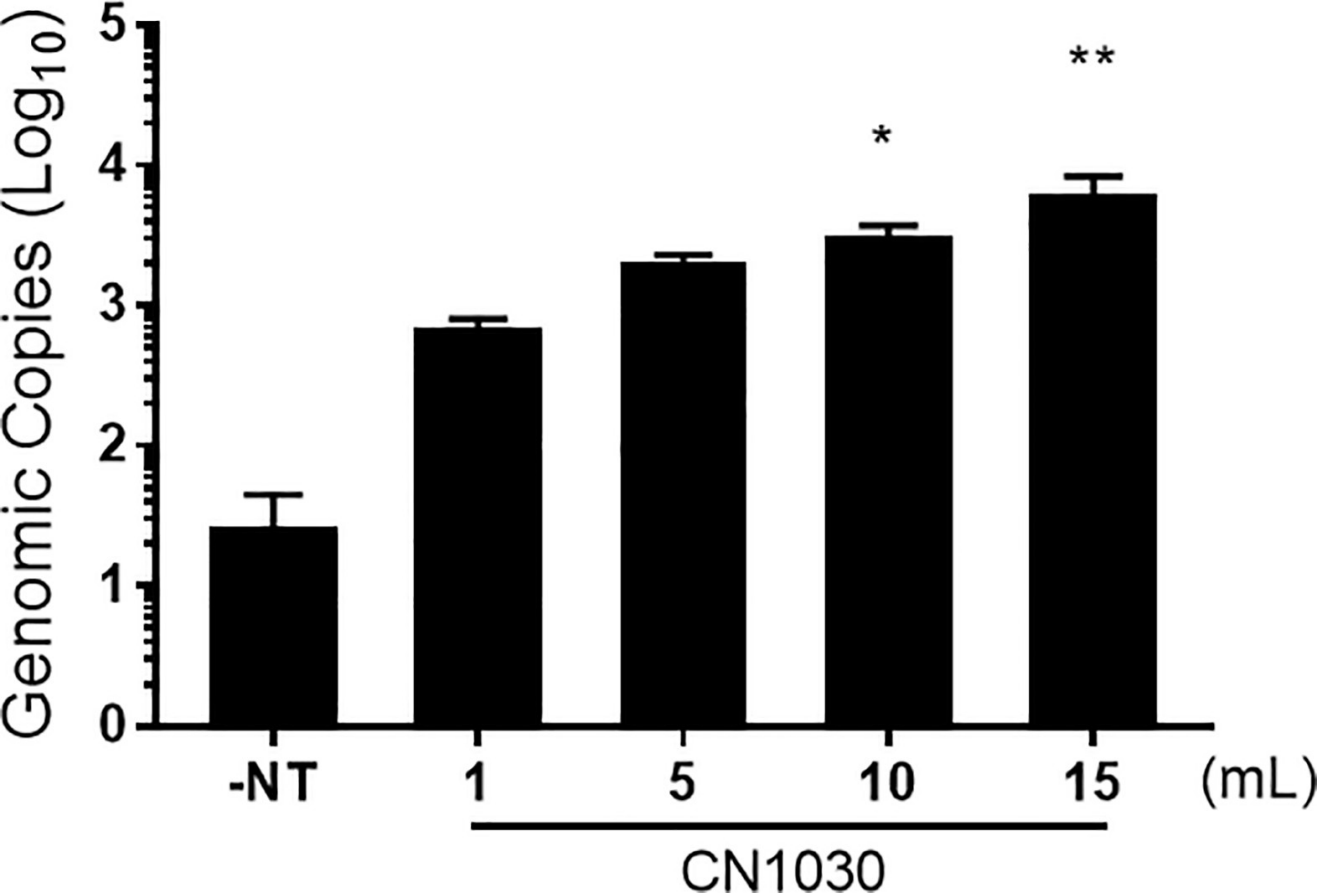
Increasing volumes of urine enhanced ZIKV RNA in urine by CN1030. ZIKV was spiked into various volume of urine (10 PFU/mL) and incubated with 100 μL of CN1030 for 30 min at ambient temperature followed by quantification of viral genomic copy via RT-qPCR. Data are presented as mean ± SEM for five biological replicates, except for the 1mL samples where three biological replicates were performed. The statistical significance was demonstrated with Kruskal-Willis test. *, *P* < 0.05. **, *P* < 0.01.

A key aspect of NT particles is their ability to concentrate analytes, even from large volumes. Therefore, given that no NT particle enrichment of viral RNA was observed at the lower titers tested, we evaluated whether increasing the sample processing volume would enable enhanced detection of ZIKV. For these experiments, ZIKV was spiked into 1, 5, 10, and 15 mL of urine at a final concentration of 10 PFU/mL and NT particle capture evaluated by RT-qPCR. Significant enhancement of ZIKV detection was observed with the 10- and 15-mL samples (Fig 5). These data indicate processing of increased sample volumes with NT particles enables enhanced detection of ZIKV at low viral titers.

### NT magnetic particles are capable of capturing and enriching ZIKV and CHIKV

**Fig 6:**
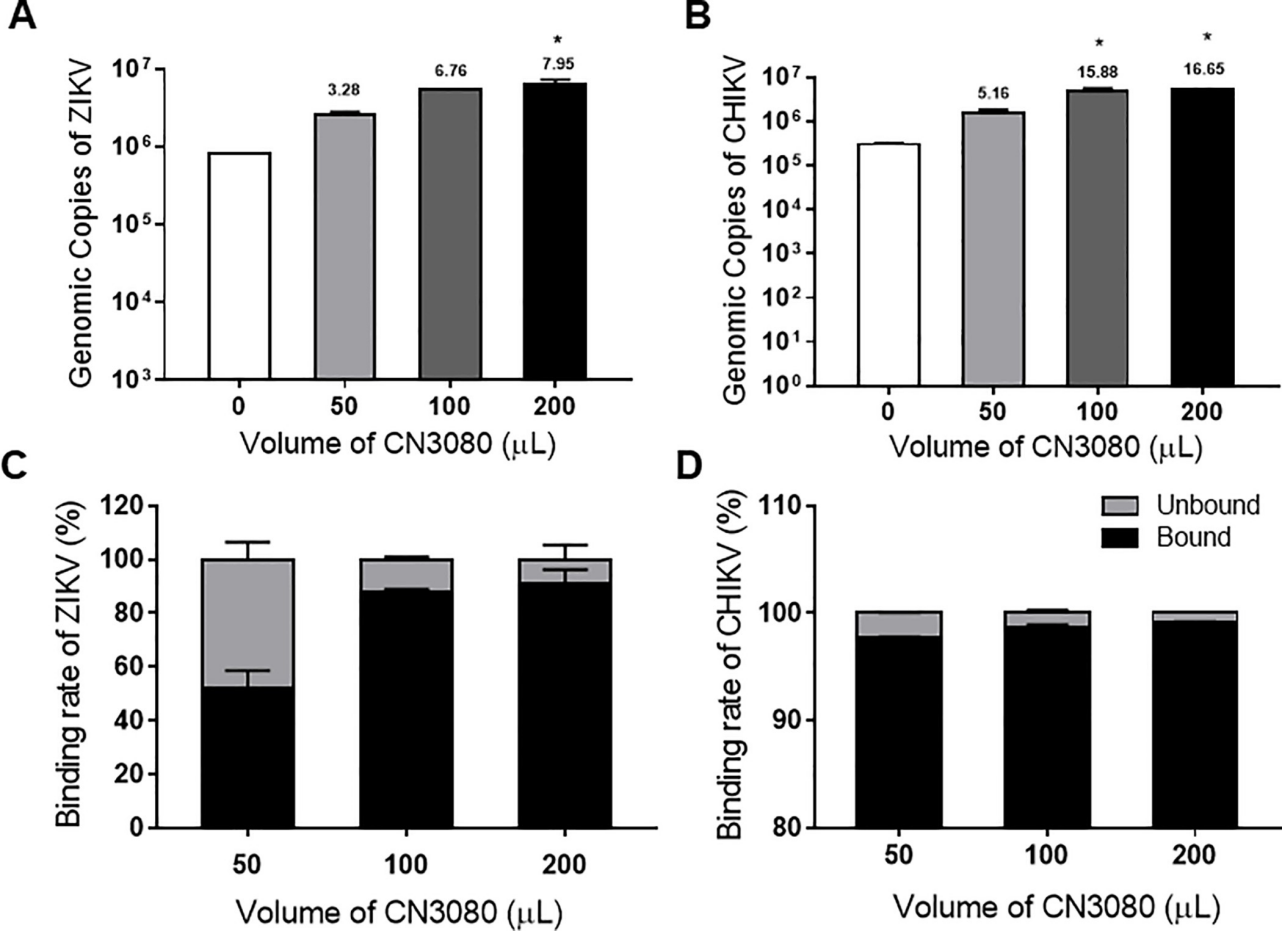
NT magnetic particles are capable of capturing and enriching ZIKV and CHIKV in urine. Different volumes of NT magnetic particle, CN3080, were incubated with ZIKV and CHIKV spiked in urine and viral capture quantified by RT-qPCR. The viral enrichment of (A) ZIKV and (B) CHIKV were determined by q-RT-PCR. The binding efficacies of CN3080 were calculated based on the unbound (C) ZIKV and (D) CHIKV RNA in the supernatant after CN3080 incubation. Data represents results from three biological replicates as mean ± SD. The statistical significance was determined by comparing each +NT group with its -NT group with Kruskal-Wallis Test where * indicates p value is ≤ 0.05.

In an attempt to shorten the processing time and simplify the NT particle workflow, CN1030 was modified by coating the particle with a magnetic shell (CN3080) which allowed it to be easily separated when loaded on a magnetic base. Different volumes of CN3080 (50, 100, and 200 μL) were tested with ZIKV and CHIKV to determine if CN3080 could enrich the viruses in a similar manner as CN1030. Following the previous experimental protocol, except magnetic separation was substituted for centrifugation, both viruses were significantly enriched by CN3080 via RT-qPCR detection in a dose-dependent manner (Fig 6A and 6B). Specifically, 100 μL and 200 μL of CN3080 yielded the greatest enrichment of both CHIKV and ZIKV. A 16-fold enrichment of CHIKV in urine by CN3080 was observed while CN3080 yielded 7-fold enrichment of ZIKV when compared samples with NT particles. These data indicate that next-generation magnetic NT particles can enhance CHIKV and ZIKV detection. Furthermore, when we analyzed the binding efficacy of CN3080 to ZIKV and CHIKV, we found that CN3080 could reach to nearly 90% of binding rate to capture ZIKV virion at 100 and 200 μL. Contrarily, the binding rate of CN3080 to CHIKV attained to over 95% even with the lowest volume of 50 μL, suggesting the CN3080 appeared to exhibit strong affinity to capture CHIKV (Fig 6C and 6D).

### Protein-ligand prediction modeling of NT particle affinity baits to viral envelope proteins

To better understand the interaction between viral envelope proteins and the affinity baits in our study, we conducted protein-ligand docking prediction with SwissDock website by using CHIKV envelope glycoprotein heterodimer [24] (PDB ID: 3N43) and ZIKV E homodimer protein [25] (PDB ID: 5JHM) along with each affinity baits (cibacron blue, reactive red 120, and acrylic acid). The SwissDock estimated free energy of binding (ΔG) towards CHIKV envelope glycoproteins for the best docked conformations was -9.53 kcal/mol for cibacron blue, -8.99 kcal/mol for reactive red 120 and -5.05 kcal/mol for acrylic acid. Similarly, the estimated free energy of binding to the ZIKV envelope protein was -8.72, -9.36, and -5.26 kcal/mol, respectively, as shown in the visualized model displays via UCSF Chimera software in S1 Fig [20], indicating that reactive red 120 and cibacron blue have stronger binding affinities to envelope proteins of CHIKV and ZIKV than acrylic acid. Additionally, hydrogen bonds (white arrows in S1 Fig) were detected by UCSF Chimera software using a geometrical definition, suggesting an importance in stabilizing the interaction between chemical baits and viral glycoproteins. These docking predictions are supportive of reactive red 120 having a strong interaction with the viral envelope proteins; however they do not take into account additional NT particle interactions that may contribute to CN1030 being the most efficient NT particle for ZIKV and CHIKV enrichment.

## Discussion

Our study addresses the ability of NT particles to serve as a pre-diagnostic processing platform to enhance RT-qPCR detection of various arboviruses causing febrile illness. We screened and assessed the fold enrichment of CHIKV, ZIKV, and DENV for 4 different NTs, containing various chemical baits and demonstrated that CN1030 with reactive red 120 affinity bait could effectively capture all tested viruses and enrich the detection sensitivity (Fig 1). This enrichment was observed even when viruses were mixed in a human fluid containing more than one virus (Fig 2). However, the enrichment observed for individual viruses were different. One possible explanation for these results is that the constitutes of the envelope structures between alphavirus (CHIKV) and flavivirus (ZIKV) are fundamentally different; distinct viral glycoproteins may have different affinities for the NT particles conferring differentiated enrichment. We also found that CN1030 substantially sustained ZIKV infectious virions in saliva detectable via conventional plaque assays for up to 72 hours (Fig 3). Viral detection by RT-qPCR was examined at multiple viral titers, with NT particles enhancing detection as low as 10^2^ PFU/mL for ZIKV and 10^1^ PFU/mL CHIKV (Fig 4). Additionally, we found for the first time that a magnetic version of CN1030, CN3080, exhibited comparable capability for capture and enrichment of ZIKV and CHIKV (Fig 6) and thus can be used to simplify the NT sample processing workflows. These particles can be integrated into existing liquid handling systems for automated and high-throughput pre-concentration and extraction prior to analysis. Additionally, we expect that magnetic CN1030 particles (CN3080) can be integrated directly into sample collection and simple processing devices suitable for use in remote or point-of-care settings.

Urine and saliva are useful clinical samples for ZIKV detection, given that ZIKV can be found at higher levels in both saliva and urine as compared to serum [26, 27]. However, viral RNA levels are still quite low, with urine samples from the 2016 ZIKV outbreak in Brazil containing 3.66–3568.61 copies [28]. CHIKV can also be found in saliva and urine [29, 30], but use of these samples did not enhance the detection rate [29]. In our studies, NT particle enhancement of ZIKV and CHIKV was not observed in urine samples at low viral titers (10 and 1 PFU/mL for ZIKV and 1 PFU/mL for CHIKV). Therefore, the use of NT particles in clinical practice will require more extensive optimization of work flows to enable viral detection in samples containing low viral genomic copies. One way this can be at least partially addressed is through processing of larger sample volumes. Urine is extremely amendable to this given the large volume that can be obtained. We found that ZIKV detection was enhanced with NT particles using larger volume samples (Fig 5). On the opposite end of the spectrum, viruses are readily detectable in clinical samples that contain high amounts of viral RNA and thus NT particles are not needed for these samples. However, it should be noted that NT particle addition did not hinder viral detection at higher viral titers and can contribute to viral stability (Fig 3). Nevertheless, the enrichment of virus by NT particles should be carefully considered in any diagnostic workflow to ensure accurate quantitation of viral genomic copies as overwhelming viral concentrations might lead to the underestimation of the amount of viral RNA present in the sample.

The structure of NT particles applied in our study is composed of an N-isopropylacrylamide core with a diameter of roughly 300 nm, affinity baits associated with the inner core, and a hydrogel VSA shell for CN2010 and CN2030 to sieve highly abundant proteins with high molecular weights, like albumin in the serum. Our data reveals that CN1030 without the VSA shell exhibited the most abundant enrichment for capturing arboviruses. A previous study exploiting NT for capturing HIV could explain our results. Small HIV proteins like Tat, Nef, and GP41 were found to easily enter through the shell and be captured by the affinity baits attached to the inner core while larger HIV virions (100–120 nm) could be sequestered and stay in the shell [5]. Flaviviruses, like ZIKV and DENV, are much smaller than HIV with an approximate diameter of 40–43 and 48–50 nm, respectively [31, 32], while CHIKV is about 65 nm in diameter [33]. Presumably, the viruses in our study could more feasibly access the affinity baits in NT particles without a shell, which may facilitate enrichment.

Another explanation for CN1030 being more suitable for viral enrichment is the properties of reactive red 120. Reactive red 120 has been used for dying cotton and cellulose fiber in textile industries for years due to its chemical and photo stable polymetric amine composition [34]. A recent study further showed that pH could change the number of binding sites of reactive red 120. That is, when the pH of reactive red 120 decreases, the number of positively charged amine groups increase [35]. Since we employed human urine fluid which is about pH 6 [36], this acidic environment could possibly enhance the interaction between viruses and reactive red 120. Also, this pH variation was not reflected in our structural docking models which needs to be considered when interpreting the protein-ligand docking results.

Co-infection of multiple arboviruses is becoming a more serious issue, especially in South and Central America where different mosquito species circulate in the same area such as *Aedes aegypti* and *Aedes albopictus*, both of which are major vectors for transmitting CHIKV, DENV and ZIKV. The geographical distribution of these two mosquitos highly overlap [37, 38]. It is necessary to have a universal tool like NT particles to enhance the detection capability of current methods for these patients in the co-circulating area. Despite the successful results in this study, it is notable that we simply evaluated the capturing efficiency of NT in our co-infection models by spiking viruses into urine, and this has to be validated for clinical samples potentially containing multiple infectious arboviruses.

## Supporting information

S1 Fig Docking prediction of ZIKV and CHIKV envelope proteins and affinity baits. The prediction modeling was generated by SwissDock using native binding modes and the predicted clusters were ranked by average FullFitness of input elements including estimated free energies of binding. The protein structures were visualized via UCSF Chimera software v.1.13.1, displayed in ribbon mode where colors in the structures indicate different chains. The green lines connecting the affinity baits and proteins are the locations of predicted hydrogen bonds by UCSF Chimera software, pointed out by white arrows. ΔG values are reported by SwissDock.
(TIF)
