# Supplementary figures and images for: Use of Nanotrap particles for the capture and enrichment of Zika, chikungunya and dengue viruses in urine
Source: PLoS One. 2020 Jan 7;15(1):e0227058. doi: 10.1371/journal.pone.0227058 (PMC6946132; doi:10.1371/journal.pone.0227058)

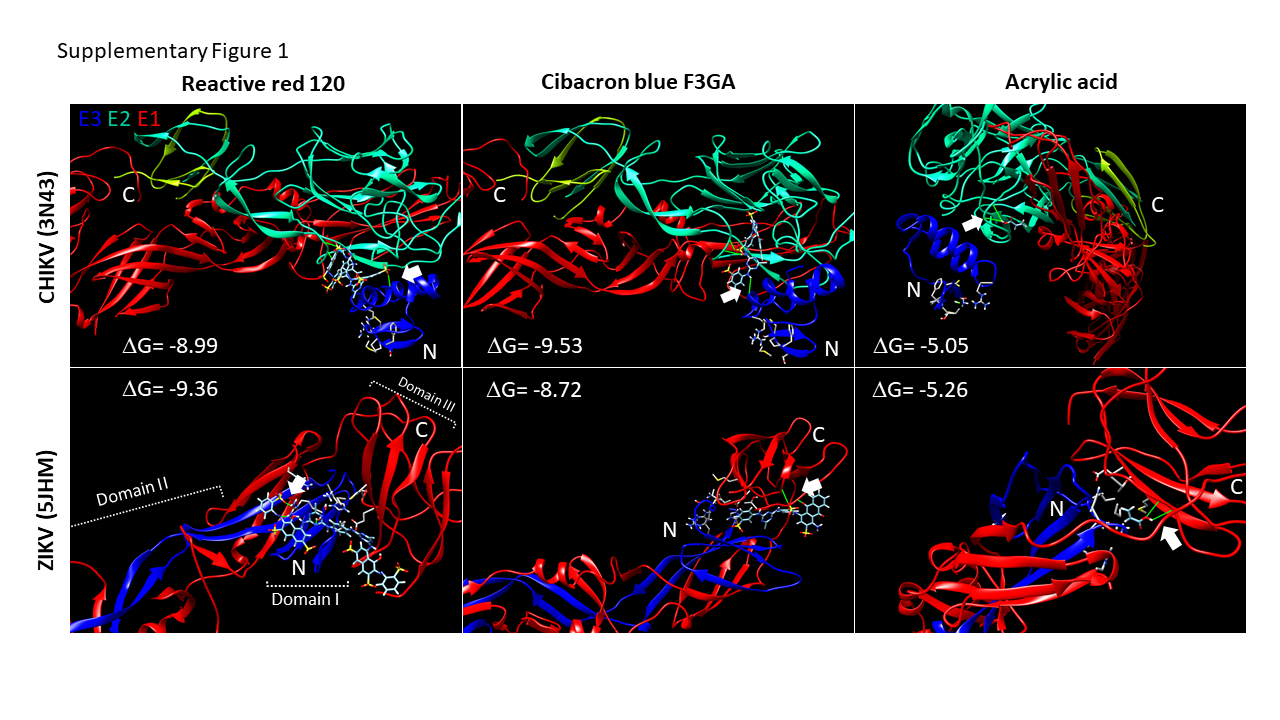

Supplement: S1 Fig — The prediction modeling was generated by SwissDock using native binding modes and the predicted clusters were ranked by average FullFitness of input elements including estimated free energies of binding. The protein structures were visualized via UCSF Chimera software v.1.13.1, displayed in ribbon mode where colors in the structures indicate different chains. The green lines connecting the affinity baits and proteins are the locations of predicted hydrogen bonds by UCSF Chimera software, pointed out by white arrows. ΔG values are reported by SwissDock. (TIF) [file pone.0227058.s001.tif]
